# First telomere-to-telomere gapless assembly of the rice blast fungus *Pyricularia oryzae*

**DOI:** 10.1038/s41597-024-03209-z

**Published:** 2024-04-13

**Authors:** Zhigang Li, Jun Yang, Xiaobei Ji, Jintao Liu, Changfa Yin, Vijai Bhadauria, Wensheng Zhao, You-Liang Peng

**Affiliations:** 1 MARA Key Laboratory of Pest Monitoring and Green Management, College of Plant Protection, China Agricultural University, Beijing, 100193 China; 2 Sanya Institute of Breeding and Multiplication/School of Tropical Agriculture and Forestry, Hainan University, Haikou, 570228 China

**Keywords:** Pathogens, Fungal genomics

## Abstract

Rice blast caused by *Pyricularia oryzae* (syn., *Magnaporthe oryzae*) was one of the most destructive diseases of rice throughout the world. Genome assembly was fundamental to genetic variation identification and critically impacted the understanding of its ability to overcome host resistance. Here, we report a gapless genome assembly of rice blast fungus *P. oryzae* strain P131 using PacBio, Illumina and high throughput chromatin conformation capture (Hi-C) sequencing data. This assembly contained seven complete chromosomes (43,237,743 bp) and a circular mitochondrial genome (34,866 bp). Approximately 14.31% of this assembly carried repeat sequences, significantly greater than its previous assembled version. This assembly had a 99.9% complement in BUSCO evaluation. A total of 14,982 genes protein-coding genes were predicted. In summary, we assembled the first telomere-to-telomere gapless genome of *P. oryzae*, which would be a valuable genome resource for future research on the genome evolution and host adaptation.

## Background & Summary

*Pyricularia oryzae* (syn., *Magnaporthe oryzae*), an ascomycete fungal pathogen, causes rice blast, one of the most destructive diseases of rice throughout the world^1,2^. The pathogen is an important and long-established model species for understanding fungal-plant interactions^3,4^. Previously, we sequenced and assembled the first genomes of field strains (P131 and Y34) and performed a comparative analysis between the laboratory and field strains, which demonstrated that translocation of transposable elements (TEs), gain or loss of isolate-specific genes and gene family expansion are essential factors, delimiting genomic plasticity and adaptability of *P. oryzae*^5^. Although these assemblies had facilitated the understanding of the genome characteristics of *P. oryzae*, the genome of the two strains were highly fragmented to more than one thousand scaffolds, for Sanger (2-fold) and 454 (18-fold) sequencing technologies were used in the previous study. Recently, over 50 genomes of different strains of *P. oryzae* have been available in public genome databases. These genomes were sequenced on the next-generation sequencing platforms, such as second-generation sequencing platforms (e.g., Illumina sequencers) and/or third-generation sequencing platforms [e.g., Pacific Biosciences (PacBio)], which facilitated the genetic studies of genomic changes and pathogenicity variation within *P. oryzae*^6–8^. However, currently most of these assemblies are fragmented and contain a large number of unplaced contigs and/or gaps owing to the presence of repetitive DNA elements in the *P. oryzae* genomes, which prevented the dissection of molecular mechanisms of adaptive evolution. Since the importance of genome assembly completeness in genomic analysis, we re-assemble the genome of *P. oryzae* stain P131 by combining Illumina, PacBio sequencing and high throughput chromatin conformation capture (Hi-C) mapping, which was the first telomere-to-telomere gapless assembly of the *P. oryzae* genome.

**Fig. 1:**
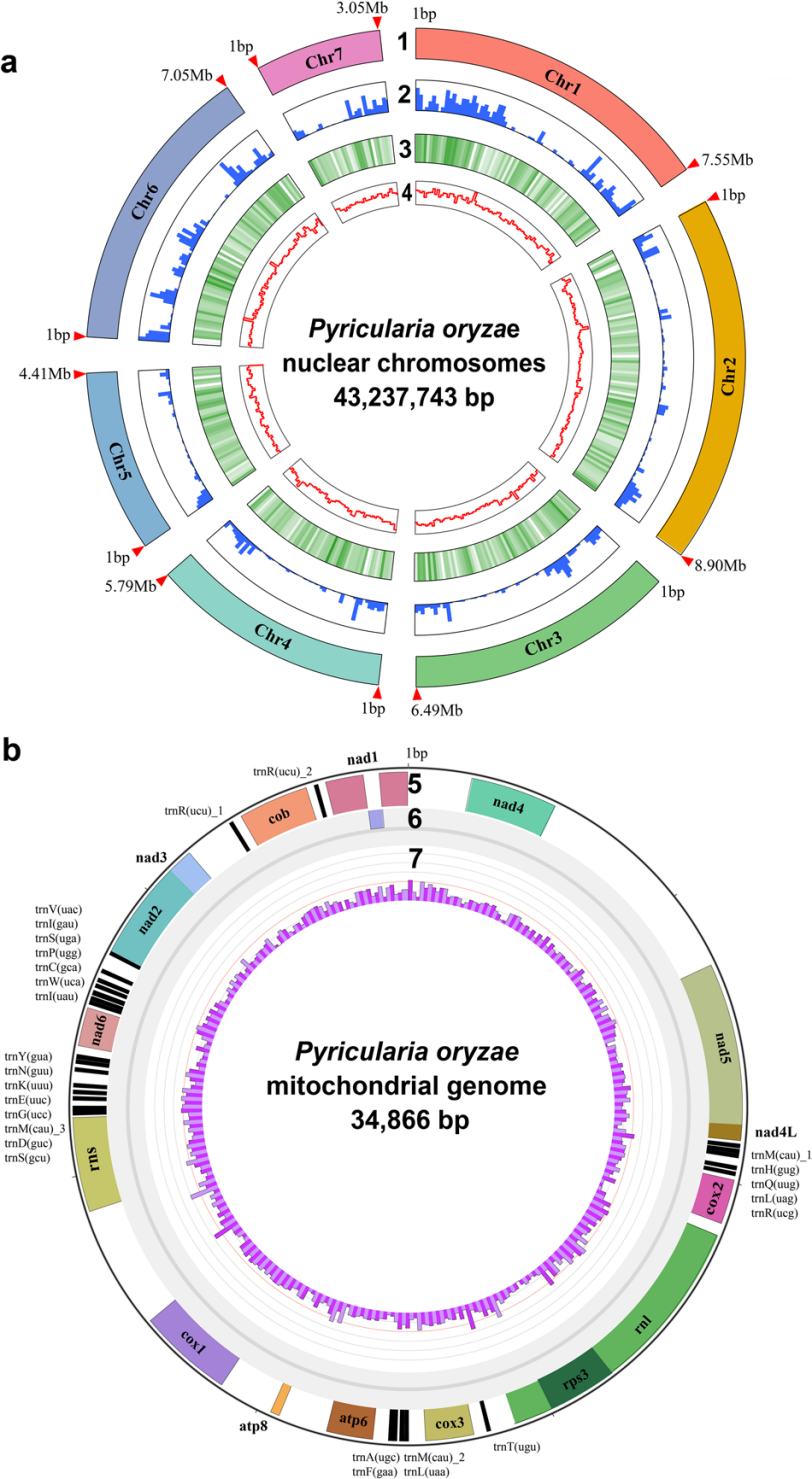
The *P. oryzae* strain P131 genome assembly. (**a**) Nuclear genome: Track 1 illustrates the seven assembled nuclear chromosomes with the indicated sizes. The red arrows at the end of chromosomes indicate telomeric repeat sequences (TTAGGG)_n_. Tracks 2 through 4 show transposon distribution, gene density, and GC density on the seven chromosomes, respectively. (**b**) Mitochondrial genome: Track 5 depicts a circular illustration of the mitochondrial genome carrying genes (represented by different color blocks), including genes encoding 14 standard fungal core PCGs (*nad1*-*nad6*, *nad4L*, *cob*, *cox1*-*cox3*, *atp6*, *atp8*, and *rps3*), ribosomal subunits (*rns* and *rnl*) and 27 tRNA genes. Tracks 6 and 7 display the introns present in the above genes and GC density of the mitochondrial genome, respectively.

**Fig. 2:**
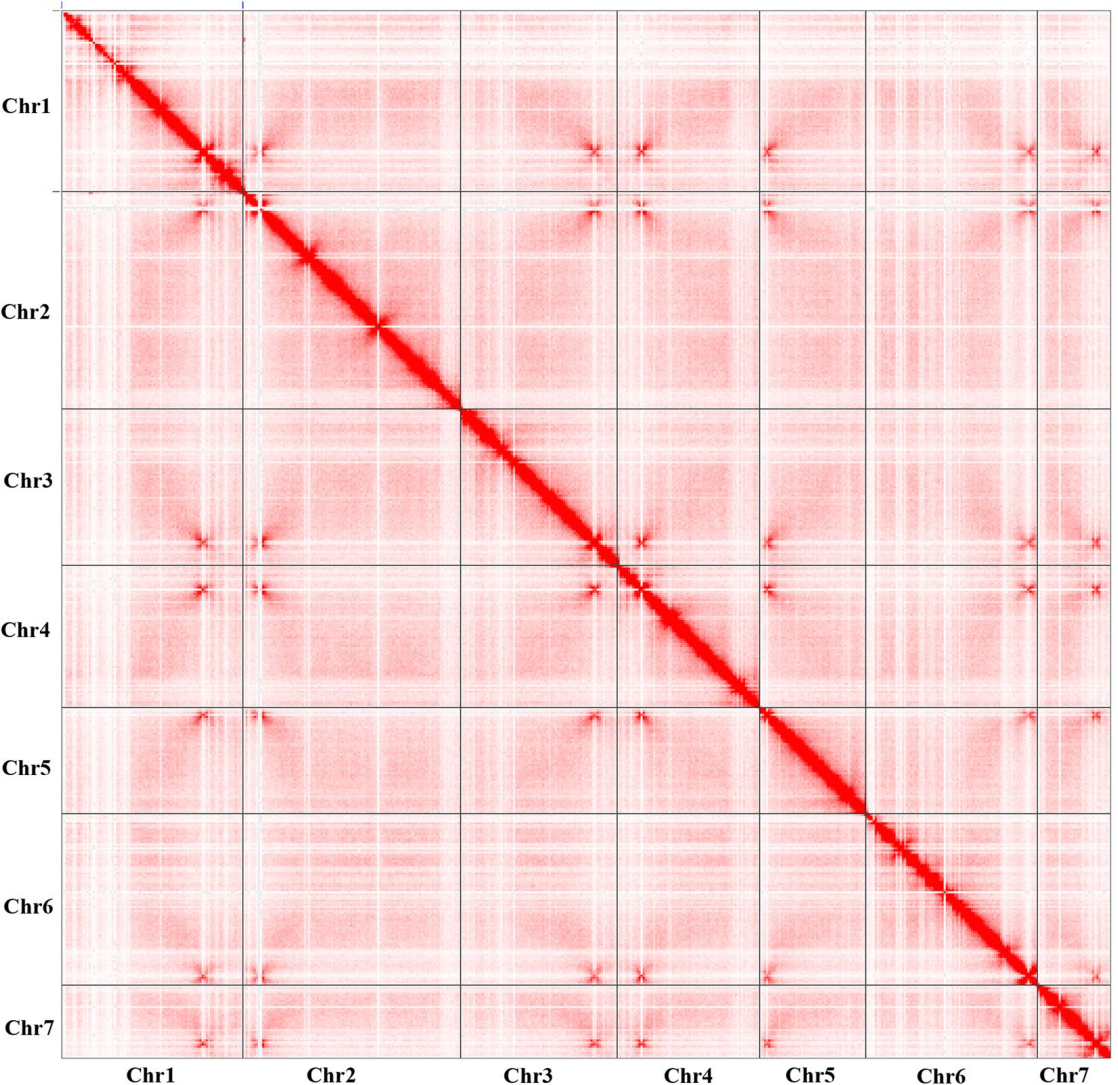
An illustration of Hi-C genome-wide interaction map result.

**Fig. 3:**
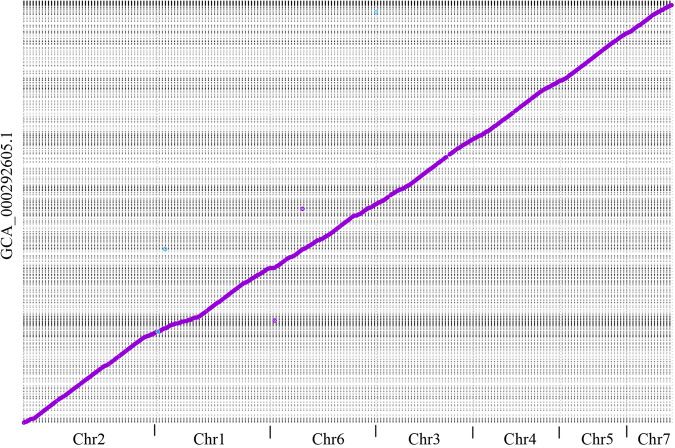
The alignment of scaffolds from the previous assembly to the new assembled chromosomes. The X and Y axis represented the new chromosomes and previous scaffolds, respectively.

A total of 10.03 Gb PacBio long-read sequencing data (~250x genome coverage) and 4.44 Gb Illumina short-read sequencing data were generated (Table 1). Hi-C library was prepared, sequenced and generated 5.57 Gb sequencing data (~140x genome coverage). The long reads were *de novo* assembled and corrected. The short reads were used to polish the assembly. Redundant genomic contigs or mitochondrial contigs were then removed. The Hi-C sequencing data were used to anchor and refined remained contigs. The mitochondrial genome was assembled independently by Mitochondrial Long-read Iterative Assembly (MLIA) pipeline^9^. The final polishing of the complete genome was performed. Finally, seven gapless chromosomes (43,237,743 bp with a contig N50 of 7.05 Mb; Fig. 1a) and a circular mitochondrial genome (34,866 bp; Fig. 1b) were constructed in the final assembly (Fig. 2). The new assembly represented a significant improvement over the previous version GCA_000292605.1^5,10^ (1,823 assembled contigs and contig N50 = 12.3 kb; see Table 2 and Fig. 3).

**Table 1:** Summary of sequencing raw data of *P. oryzae* strain P131.

Statistics	PacBio	Hi-C	IIlumina	Total
Library size (bp)	15,000	350	350	—
Raw data (Gb)	10.03	5.57	4.44	20.04
N50 (bp)	14,456	150	150	—
Mean read length (bp)	12,511	150	150	—
Coverage (X)	250	140	110	500

**Table 2:** Summary of the genome assembly.

Statistics	GCA_000292605.1	This study
Assembly size (bp)	37,955,031	43,237,743
GC content (%)	51.10	51.12
Number of scaffolds	1,822	7
Scaffold N50 (bp)	62,822	7,048,921
Max scaffold (bp)	459,401	8,902,985
Gap number	843	0
Gap length (bp)	269,429	0
Number of genes	12,713	14,982
Number of transcripts	12,713	20,811
BUSCO^a^	98.2% (744/6)	99.9% (757/1)
BUSCO^b^	97.6% (1665/28)	99.4% (1696/10)

^a^using “fungi_odb10” lineage with 758 BUSCO markers; ^b^using “ascomycota_odb10” lineage with 1706 BUSCO markers. The numbers in parentheses were “complete and fragmented orthologues” and “missing orthologues”, respectively.

The nuclear genome was annotated by Braker2 pipeline^11^. The mitochondrial genome was annotated by MFannot^12^ using genetic code 4. In conclusion, the nuclear genome is predicted to contain 14,968 genes (including 20,797 transcripts), and the mitochondrial genome is likely to carry 14 conserved protein-coding genes (Table 3). A total of 99.9% of the BUSCOs were mapped onto the P131 genome assembly. Approximately 14.31% of the genome carried repeat sequences, most of which were TEs, which was significantly greater than the previous version (Table 4).

**Table 3:** Detailed summary of assembled chromosomes.

Genome	Length (bp)	Gene number	Transcript number
Chr1	7,553,526	2,662	3,668
Chr2	8,902,985	2,988	4,208
Chr3	6,487,520	2,273	3,169
Chr4	5,790,573	1,997	2,750
Chr5	4,405,643	1,536	2,092
Chr6	7,048,921	2,489	3,489
Chr7	3,048,575	1,023	1,421
Mitochondria	34,866	14	14
Total	43,272,609	14,982	20,811

**Table 4:** Classification of repeat sequences.

Type (bp/number)	GCA_000292605.1	This study	Difference (%)
LINEs	36,081/102	803,175/305	+2126.0/199.0
LTR elements	495,032/716	3,133,473/1,622	+533.0/126.5
DNA transposons	65,073/278	1,033,513/805	+1488.2/189.6
Unclassified interspersed repeats	350,30/1,885	686,834/2,449	+96.1/29.9
Simple repeats	410,209/11,056	452,786/12,188	+10.4/10.2
Low complexity	65,698/1,538	68,573/1,550	+4.4/0.8

The telomere repeat sequence (TRS) (TTAGGG)_n_ was presented on both ends of chromosomes 2, 4, 5, 6, and 7 and one end of chromosomes 1 and 3 in our assembly. We then compared the TRS in the published near-complete assembled genome of *P. oryzae* strains with the genome assembly generated in this study. Interestingly, minority deficiency and telomere variability of TRSs in *P. oryzae* were extensively observed, which may play subtle roles in pathogenic adaptation^13–15^. In summary, we assembled the first telomere-to-telomere gapless genome of *P. oryzae*, which can be instrumental in understanding the genome evolution and host adaptation in the rice blast fungus.

## Methods

### Sampling and DNA extraction

The *P. oryzae* strain P131 was grown and maintained on oatmeal tomato agar (OTA) plates^16^. Conidia were produced on OTA plates and harvested from 7-day culture plates grown at 25 °C under constant fluorescent light. Hyphae were collected from 2-day-old cultures in complete medium shaken at 150 rpm at 25 °C. Genomic DNA extracted from vegetative mycelia using cetyltrimethylammonium bromide (CTAB) protocol was used for genome sequencing^17^.

### Illumina, PacBio and Hi-C sequencing

Genome sequencing was conducted on Pacific Biosciences Sequel (PacBio, Menlo Park, CA) at CapitalBio Technology Co., Ltd (Beijing, China). Qualified genomic DNA was fragmented with G-tubes (Covaris, Woburn, MA, USA) and end-repaired to prepare SMRTbell DNA template libraries (with fragment size of >10 kb selected). Library quality was detected by Qubit dsDNA HS Assay Kit (Thermo Fisher Scientific, Waltham, MA, USA, Q33230). The average fragment size was estimated on Bioanalyzer 2100 (Agilent, Santa Clara, CA). SMRT sequencing was performed on the Pacific Biosciences RSII sequencer (PacBio, Menlo Park, CA) according to standard protocols using the P4-C2 chemistry. A total of 10.03 Gb PacBio sequencing data with a subread N50 of 14.5 kb. In addition, Illumina HiSeq X Ten sequencer using paired-end technology was also used to perform genome sequencing and 4.44 Gb sequencing data (150 bp paired-end reads) were yielded at CapitalBio Technology Co., Ltd (Beijing, China).

Hi-C library was prepared from cross-linked chromatins of fungal mycelia by Novogene Co., Ltd (Beijing, China). In brief, the tissue was ground and then cross-linked with 4% formaldehyde solution. After the sample of crosslinking reaction and cell lysis, nuclei were digested with 4-cutter restriction enzyme *Dpn*II. Subsequently, ligated DNA was purified and fragmented into 300 bp size on average. The constructed Hi-C library was sequenced by Illumina NovaSeq 6000. 5.57 Gb paired-end sequencing data (150-bp length) were generated. The Hi-C maps from raw data were performed by Juicer (v1.6)^18^, followed by using a manually correction with Juicebox (v2.13.07)^19^.

### RNA sequencing and analysis

Total RNA was extracted from conidia and hyphae with the Trizol reagent (Invitrogen, Carlsbad, CA, USA, 15596026) and then enriched by RNeasy Pure mRNA Bead Kit (Qiagen, Germany), respectively. High-throughput cDNA libraries were prepared according to the Illumina whole transcriptome library preparation protocol and sequenced on the Illumina GA platform by the BGI Genomics (Shenzhen, China)^20^. Quality control was performed by FastQC (v0.11; https://github.com/s-andrews/FastQC). RNA-Seq data were mapped to *P. oryzae* by HISAT2 (v2.2.1)^21^, and SAMTools (v1.12)^22^ were used to evaluate read alignments.

### Genome assembly

The *de novo* long-read assembler Canu v2.1.1^23^ (parameters: genomeSize = 44 m corOutCoverage = 200 corMinCoverage = 2 minReadLength = 4000 minOverlapLength = 800 correctedErrorRate = 0.050) was used to assemble PacBio reads to generated draft contigs, which were then corrected by GCpp v1.9 (https://github.com/PacificBiosciences/gcpp; parameters:–algorithm = arrow -x 5 -X 200 -q 40) using PacBio long-reads. The polishing step was performed by Pilon v1.23^24^ (parameters:–changes–vcf) using the Illumina short reads. Contigs were considered redundant if they aligned concordantly (identity >99%) with another contig, and the redundant contigs, along with mitochondrial contigs, were removed, resulting in a total of 13 contigs. The Hi-C sequencing data were used to anchor all 13 contigs using Juicer v1.6^18^, resulting in 7 scaffolds, which were further refined using Juicebox v2.13.07^19^. Gaps within the scaffolds were filled using LR_Gapcloser^25^. We then manually checked whether long reads aligned the bridging cross the gaps, or whether overlapping contig ends (>20 kb length and 99.9% sequence identity) existed.The mitochondrial genome was assembled independently by Mitochondrial Long-read Iterative Assembly (MLIA) pipeline^9^. The final polishing of the complete genome was performed again using Pilon v1.23^24^.

### Gene model and function annotations

Repetitive sequences of *P. oryzae* strain P131 was firstly *de novo* identified via RepeatModeler (v2.0.1)^26^ and masked by RepeatMasker (v4.1.1)^27^ (parameters: -e rmblast -pa 30 -xsmall -nolow -norna -gff -a). The nuclear genome was annotated by Braker2 pipeline^11^ (parameters: –softmasking –gff3 –fungus –gth2traingenes –prg = gth), combining three aspects evidences: *ab initio* prediction, homologous proteins, and RNA-Seq evidences. The AUGUSTUS v3.4.0^28^ and Genemark-EP+^29^ was used as *ab initio* prediction tools in the pipeline. All proteins of the genus *Pyricularia* in the Uniref100 database^30^ were collected and the 100% non-redundant protein dataset was built by cd-hit^31^ (parameters: -c 1.00 -aS 1.00 -aL 1.00 -n 5 -M 20000), which was used as the protein-based training evidence. The GenomeThreader v1.7.3^32^ was used as the alignment tool. RNA-Seq data previous used^20^ (i.e. SRR15170638^33^, SRR15170637^34^ and SRR15170636^35^) were aligned by HISAT2 (v2.2.1)^21^ (parameters: -t -dta). The mitochondrial genome was annotated by MFannot^12^ with genetic code 4.

## Data Recodes

The raw genomic sequencing data used and/or analyzed during the current study are available at NCBI Sequence Read Archive database (Accession number SRR24890910^36^, SRR24890911^37^ and SRR24890912^38^). The assembled genome was deposited under the same BioProject with *P. oryzae* strain P131 at NCBI (Accession number: GCA_000292605.2^39^; BioProject ID: PRJNA82693; BioSample ID: SAMN31867770). The accession numbers from Chr1 to Chr7 chromosome sequences were CP114135 to CP114141, respectively. And the accession number corresponding to the mitochondrial genome sequence was CP114142.

## Technical Validation

### DNA sample quality

The DNA quality was detected using Qubit (Thermo Fisher Scientific, Waltham, MA) and Nanodrop (Thermo Fisher Scientific, Waltham, MA).

### Sequencing data assessment

The short read data were assessed by fastp v0.23^40^. The genomic short sequencing reads had 49.75% GC content. The Q20 and Q30 percentages were 97.1% and 92.06%, respectively. The Hi-C sequencing data had 50.5% GC content, and had quality scores of 97.67% (Q20) and 93.64% (Q30), respectively.

### Evaluation of the genome assembly

The genome assembly quality was evaluated through the Benchmarking Universal Single-Copy Orthologs (BUSCO) tool with the “fungi_odb10” lineage as a reference dataset. The results showed that 99.9% of all 758 BUSCO markers were assembled, implying a high level of completeness of the assembly. In addition, the results generated from “ascomycota_odb10” lineage showed 99.4% of all 1706 BUSCO markers were include (Table 2).

## Data Availability

The published softwares used in this work were cited in the Methods section. If no detailed parameters were mentioned for the software, default parameters were applied.
